# Robertsite, Ca_2_Mn^III^_3_O_2_(PO_4_)_3_·3H_2_O

**DOI:** 10.1107/S160053681203735X

**Published:** 2012-09-15

**Authors:** Marcelo B. Andrade, Shaunna M. Morrison, Adrien J. Di Domizio, Mark N. Feinglos, Robert T. Downs

**Affiliations:** a Department of Geosciences, University of Arizona, 1040 E. 4th Street, Tucson, Arizona 85721-0077, USA; b Duke University Medical Center, Box 3921, Durham, North Carolina 27710, USA

## Abstract

Robertsite, ideally Ca_2_Mn_3_O_2_(PO_4_)_3_·3H_2_O [calcium manganese(III) tris­(orthophosphate) trihydrate], can be associated with the arseniosiderite structural group characterized by the general formula Ca_2_ *A*_3_O_2_(*T*O_4_)_3_·*n*H_2_O, with *A* = Fe, Mn; *T* = As, P; and *n* = 2 or 3. In this study, single-crystal X-ray diffraction data were used to determine the robertsite structure from a twinned crystal from the type locality, the Tip Top mine, Custer County, South Dakota, USA, and to refine anisotropic displacement parameters for all atoms. The general structural feature of robertsite resembles that of the other two members of the arseniosiderite group, the structures of which have previously been reported. It is characterized by sheets of [MnO_6_] octa­hedra in the form of nine-membered pseudo-trigonal rings. Located at the center of each nine-membered ring is a PO_4_ tetra­hedron, and the other eight PO_4_ tetra­hedra sandwich the Mn–oxide sheets. The six different Ca^2+^ ions are seven-coordinated in form of distorted penta­gonal bipyramids, [CaO_5_(H_2_O)_2_], if Ca—O distances less than 2.85 Å are considered. Along with hydrogen bonding involving the water mol­ecules, they hold the manganese–phosphate sheets together. All nine [MnO_6_] octa­hedra are distorted by the Jahn–Teller effect.

## Related literature

For information on the arseniosiderite group minerals, see: Moore & Ito (1974 ▶); Moore & Araki (1977 ▶); van Kauwenbergh *et al.* (1988 ▶); Voloshin *et al.* (1982) ▶. For details of sailaufite, see: Wildner *et al.* (2003 ▶). For studies on pararobertsite, see: Roberts *et al.* (1989 ▶); Kampf (2000 ▶). For research involving Mn^3+^ pairing in phosphate minerals, see: Fransolet (2000 ▶). For information on crystalline manganese phosphate-based adsorbers, see: Kulprathipanja *et al.* (2001 ▶).

## Experimental

### 

#### Crystal data

Ca_2_Mn_3_O_2_(PO_4_)_3_·3H_2_O
*M* *_r_* = 615.94
Monoclinic, 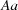

*a* = 17.3400 (9) Å
*b* = 19.4464 (10) Å
*c* = 11.2787 (6) Å
β = 96.634 (3)°
*V* = 3777.7 (3) Å^3^
*Z* = 12
Mo *K*α radiation
μ = 4.26 mm^−1^
*T* = 293 K
0.07 × 0.06 × 0.06 mm

#### Data collection

Bruker APEXII CCD area-detector diffractometer
Absorption correction: multi-scan (*SADABS*; Bruker, 2004 ▶) *T*_min_ = 0.755, *T*_max_ = 0.784
28397 measured reflections
12635 independent reflections
9678 reflections with *I* > 2σ(*I*)
*R*_int_ = 0.037

#### Refinement

*R*[*F* ^2^ > 2σ(*F* ^2^)] = 0.045
*wR*(*F* ^2^) = 0.107
*S* = 1.02
12635 reflections
677 parameters
2 restraints
Δρ_max_ = 1.74 e Å^−3^
Δρ_min_ = −1.09 e Å^−3^
Absolute structure: Flack (1983 ▶), 5755 Friedel pairs
Flack parameter: 0.676 (18)

### 

Data collection: *APEX2* (Bruker, 2004 ▶); cell refinement: *SAINT* (Bruker, 2004 ▶); data reduction: *SAINT*; program(s) used to solve structure: *SHELXS97* (Sheldrick, 2008 ▶); program(s) used to refine structure: *SHELXL97* (Sheldrick, 2008 ▶); molecular graphics: *XtalDraw* (Downs & Hall-Wallace, 2003 ▶); software used to prepare material for publication: *publCIF* (Westrip, 2010 ▶).

## Supplementary Material

Crystal structure: contains datablock(s) I, global. DOI: 10.1107/S160053681203735X/wm2672sup1.cif

Structure factors: contains datablock(s) I. DOI: 10.1107/S160053681203735X/wm2672Isup2.hkl

Additional supplementary materials:  crystallographic information; 3D view; checkCIF report

## Figures and Tables

**Table 1:** Hydrogen-bond geometry (Å)

*D*⋯*A*	*D*⋯*A*
O*w*1⋯O17	2.964 (7)
O*w*1⋯O33	2.889 (8)
O*w*2⋯O33	2.815 (7)
O*w*2⋯O*w*8	2.655 (8)
O*w*3⋯O1^i^	2.957 (4)
O*w*3⋯O17	2.821 (6)
O*w*4⋯O29	2.764 (7)
O*w*4⋯O*w*9	2.631 (8)
O*w*5⋯O*w*7^ii^	2.682 (8)
O*w*5⋯O25^iii^	2.792 (7)
O*w*6⋯O13^iv^	2.977 (6)
O*w*6⋯O*w*9^ii^	2.651 (7)
O*w*7⋯O9^v^	2.768 (7)
O*w*7⋯O*w*3^vi^	2.645 (7)
O*w*8⋯O13	2.710 (8)
O*w*8⋯O*w*1	2.635 (7)
O*w*9⋯O12^v^	2.989 (7)
O*w*9⋯O21^vii^	2.743 (8)

Symmetry codes: (i) 
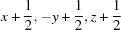
; (ii) 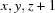; (iii) 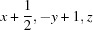; (iv) 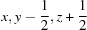; (v) 
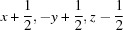
; (vi) 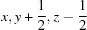; (vii) 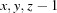.

**Fig. 1.**
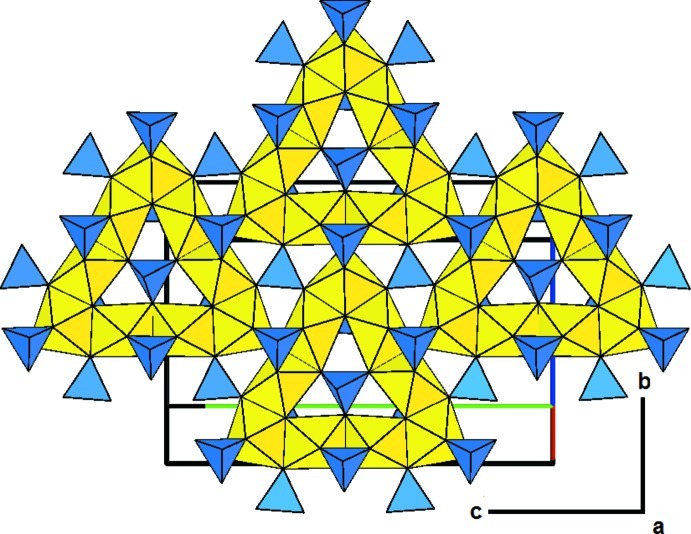
The projection of the [Mn9O6(PO4)]12 sheet in robertsite viewed down the a axis. The MnO6 octahedra and the PO4 tetrahedra are yellow and blue, respectively.

**Fig. 2.**
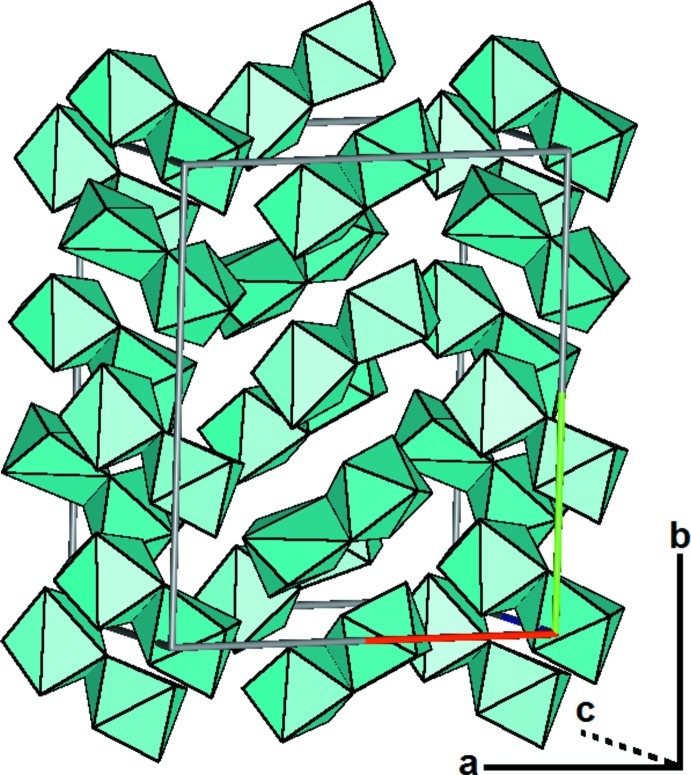
The [Ca2O10(H2O)2] dimers in the robertsite structure.

**Fig. 3.**
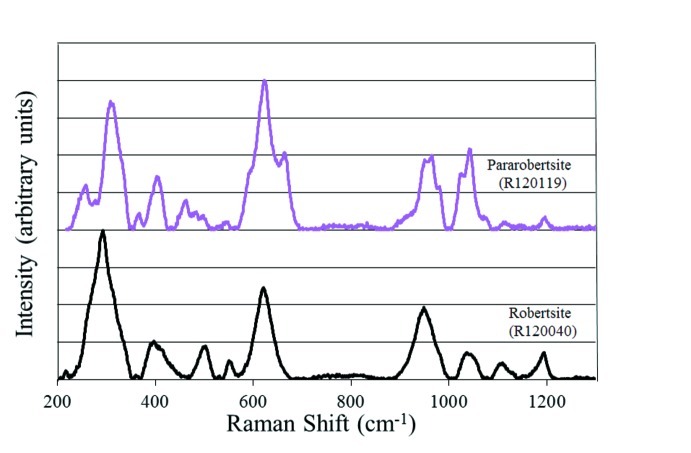
Raman spectra of robertsite and pararobertsite.

## supplementary crystallographic information

### Comment

Robertsite is a member of the arseniosiderite-type compounds adopting a sheet structure that is characterized by layers of nine-membered pseudo-trigonal rings of octahedra. They exhibit the general formula Ca_2_*A*_3_O_2_(*T*O_4_)_3_.*n*H_2_O, with *A* = Fe, Mn; *T* = As, P; and *n* = 2 or 3. There are five members of this group in the current list of minerals approved by the International Mineralogical Association (IMA), including arseniosiderite, [Ca_2_Fe^3+^_3_O_2_(AsO_4_)_3_.3H_2_O], kolfanite, [Ca_2_Fe^3+^_3_O_2_(AsO_4_)_3_.2H_2_O], mitridatite, [Ca_2_Fe^3+^_3_O_2_(PO_4_)_3_.3H_2_O], sailaufite, (Ca,Na)_2_Mn^3+^_3_O_2_(AsO_4_)_2_CO_3_.3H_2_O, and robertsite [Ca_2_Mn^3+^_3_O_2_(PO_4_)_3_.3H_2_O]. Because of ubiquitous twinning in this group, only the structures of mitridatite (Moore & Araki, 1977) and sailaufite (Wildner *et al.*, 2003) have previously been reported. Arseniosiderite, mitridatite and robertsite exhibit monoclinic symmetry with space group *Aa*. Kolfanite can also be assigned in space group *Aa*, but with weak unindexed reflections (Voloshin *et al.*, 1982). Sailaufite has been reported in space group *Cm* and a doubled cell presumably due to ordering of the carbonate group that substitutes the tetrahedral group.

Robertsite and pararobertsite (Roberts *et al.*, 1989; Kampf, 2000), are dimorphs with composition Ca_2_Mn^3+^_3_O_2_(PO_4_)_3_.3H_2_O, and are the only phosphate minerals known to date with Ca^2+^ and Mn^3+^ cations (Fransolet, 2000). They occur in altered pegmatites and sedimentary phosphate ores as typical products of weathering (van Kauwenbergh *et al.*, 1988), and are thus important to our understanding of the alteration processes of primary phosphate minerals. Crystalline manganese phosphates are also of particular interest for technological applications. For example, they have been studied as potential adsorbers of metal contaminants, such as Ag, Hg, and Pd, from industrial waste (Kulprathipanja *et al.*, 2001). Robertsite was previously investigated by Moore & Ito (1974) using powder X-ray diffraction, but its crystal structure was not refined owing to the rarity of suitable single crystals. This study presents the first crystal structure determination of robertsite. The single-crystal data was obtained from a sample from the Tip Top mine.

The structure of robertsite is built from sheets of [MnO_6_] octahedra sandwiched between layers of PO_4_ tetrahedra. The [MnO_6_] octahedra share edges to form nine-membered pseudo-trigonal rings that pack in monolayers (Fig. 1). The Mn^3+^ cations sit on their own Kagome net, as a result of the repulsion between them (Kampf, 2000). All [MnO_6_] octahedra are distorted, characteristic of the Jahn-Teller effects for high-spin Mn^3+^ with *d*^4^ electromic configuration. Among the nine [MnO_6_] octahedra, that of Mn7 is flattened, while the others are elongated. For example, the Mn1 cation is surrounded by four short equatorial O atoms at 1.967 (5), 1.992 (5), 1.956 (4), and 1.862 (4) Å and by two axial O atoms at 2.137 (4) and 2.138 (4) Å. In contrast, the two-axial O atoms, O7 and O23, bonded to Mn7 are at 1.952 (5) and 1.898 (5) Å, respectively, whereas the four equatorial O atoms are at approximately 2.05 Å. Situated at the center of each nine-membered ring is a PO_4_ tetrahedron. The sheets of [MnO_6_] octahedra and PO_4_ tetrahedra are stacked together along the *a* axis by water molecules and a double layer of Ca^2+^ cations. All Ca^2+^ cations can be described as seven-coordinated, [CaO_5_(H_2_O)_2_], in form of pentagonal bipyramidal polyhedra, if distances less than 2.85 Å are considered. Each [CaO_5_(H_2_O)_2_] polyhedron shares an edge with another one to form [Ca_2_O_10_(H_2_O)_2_] dimers, which may be the reason for the symmetry reduction from trigonal to monoclinic for this mineral (Fig. 2). In addition, one-third of the water molecules are loosely bonded in cavities of the structure. Although H atoms were excluded from the refinement, it is obvious from O···O distances that medium-strong hydrogen bonds are present in the structure. In Table 1, a possible hydrogen-bonding scheme devised from O···O distances is presented.

Figure 3 displays the Raman spectrum of robertsite, along with that of parabobertsite for comparison. Evidently, the two spectra are similar. The major Raman bands for the two minerals can be grouped into four different regions. The bands in the high-frequency region (800–1250 cm^-1^) are attributed to the P—O symmetric and asymmetric stretching modes within the PO_4_ group. The bands in the middle-frequency region (520–790 cm^-1^) originate from the O—P—O bending vibrations. The bands between 250 and 520 cm^-1^ may be ascribed to the stretching vibrations of the Mn—O bonds. The bands below 250 cm^-1^ are of a complex nature, mostly resulting from Mn–O and Ca—O interactions, lattice vibrations and librations, as well as rotational and translational motions of PO_4_. Noticeably, many Raman bands for pararobertsite are split compared to those for robertsite, related to the lowering of symmetry (*P*2_1_/*c* for pararobertsite *versus Aa* for robertsite).

### Experimental

The robertsite specimen used in this study comes from the type locality, the Tip Top mine, Custer County, South Dakota and is in the collection of the RRUFF project (deposition No. R120040; http://rruff.info). The chemical composition, Ca_1.93_(Mn_2.92_Fe_0.07_)_Σ__=2.99_O_1.84_(PO_4_)_3.04_.2.72H_2_O, from the type specimen was reported by Moore & Ito (1974).

The Raman spectra of robertsite and pararobertsite (R120119) were collected from a randomly oriented crystal at 100% power on a Thermo Almega microRaman system, using a solid-state laser with a wavenumber of 532 nm, and a thermoelectrically cooled CCD detector. The laser is partially polarized with 4 cm^-1^ resolution and a spot size of 1 µm.

### Refinement

Due to the similar scattering power of Mn and Fe, any minor Fe was treated as Mn and therefore the ideal chemical formula was assumed during the refinement. The structure, in space group *Aa*, was refined on basis of data from a crystal twinned by inversion with a ratio of 0.676 (18):0.324 (18) for the twin components. The maximum residual electron density in the difference Fourier maps was located at (0.0711, 0.2978, 0.8376), 0.71 Å from Ca2 and the minimum at (0.1865, 0.4867, 0.0185) 0.68 Å from P7. H-atoms from water molecules could not be assigned reliably and were excluded from refinement. To keep consistent with the previous report on mitridatite (Moore & Araki, 1977), the non-standard space group setting of space group No. 9 in *Aa* was adopted here, instead of the conventional *Cc* setting.

### Crystal data

**Table:**

Ca_2_Mn_3_O_2_(PO_4_)_3_·3H_2_O	*F*(000) = 3600
*M**_r_* = 615.94	pseudohexagonal
Monoclinic, *A**a*	*D*_x_ = 3.238 Mg m^−^^3^
Hall symbol: A -2ya	Mo *K*α radiation, λ = 0.71073 Å
*a* = 17.3400 (9) Å	Cell parameters from 5282 reflections
*b* = 19.4464 (10) Å	θ = 2.4–31.7°
*c* = 11.2787 (6) Å	µ = 4.26 mm^−^^1^
β = 96.634 (3)°	*T* = 293 K
*V* = 3777.7 (3) Å^3^	Block, brown
*Z* = 12	0.07 × 0.06 × 0.06 mm

### Data collection

**Table:**

Bruker APEXII CCD area-detector diffractometer	12635 independent reflections
Radiation source: fine-focus sealed tube	9678 reflections with *I* > 2σ(*I*)
Graphite monochromator	*R*_int_ = 0.037
φ and ω scan	θ_max_ = 32.6°, θ_min_ = 2.1°
Absorption correction: multi-scan (*SADABS*; Bruker, 2004)	*h* = −26→26
*T*_min_ = 0.755, *T*_max_ = 0.784	*k* = −24→29
28397 measured reflections	*l* = −17→17

### Refinement

**Table:**

Refinement on *F*^2^	Primary atom site location: structure-invariant direct methods
Least-squares matrix: full	Secondary atom site location: difference Fourier map
*R*[*F*^2^ > 2σ(*F*^2^)] = 0.045	*w* = 1/[σ^2^(*F*_o_^2^) + (0.0478*P*)^2^] where *P* = (*F*_o_^2^ + 2*F*_c_^2^)/3
*wR*(*F*^2^) = 0.107	(Δ/σ)_max_ = 0.001
*S* = 1.02	Δρ_max_ = 1.74 e Å^−^^3^
12635 reflections	Δρ_min_ = −1.09 e Å^−^^3^
677 parameters	Absolute structure: Flack (1983), 5755 Friedel pairs
2 restraints	Flack parameter: 0.676 (18)
0 constraints	

### Special details

**Table:**

Geometry. All e.s.d.'s (except the e.s.d. in the dihedral angle between two l.s. planes) are estimated using the full covariance matrix. The cell e.s.d.'s are taken into account individually in the estimation of e.s.d.'s in distances, angles and torsion angles; correlations between e.s.d.'s in cell parameters are only used when they are defined by crystal symmetry. An approximate (isotropic) treatment of cell e.s.d.'s is used for estimating e.s.d.'s involving l.s. planes.
Refinement. Refinement of *F^2^* against ALL reflections. The weighted *R*-factor *wR* and goodness of fit *S* are based on *F^2^*, conventional *R*-factors *R* are based on *F*, with *F* set to zero for negative *F^2^*. The threshold expression of *F^2^* > σ(*F^2^*) is used only for calculating *R*-factors(*gt*) *etc*. and is not relevant to the choice of reflections for refinement. *R*-factors based on *F^2^* are statistically about twice as large as those based on *F*, and *R*- factors based on ALL data will be even larger.

### Fractional atomic coordinates and isotropic or equivalent isotropic displacement parameters (Å^2^)

**Table:**

	*x*	*y*	*z*	*U*_iso_*/*U*_eq_	
Ca1	0.58062 (8)	0.03854 (7)	0.82279 (11)	0.0137 (3)	
Ca2	0.58141 (8)	0.17089 (7)	0.36808 (11)	0.0124 (3)	
Ca3	0.57448 (8)	0.37140 (7)	0.83846 (13)	0.0179 (3)	
Ca4	0.41585 (9)	0.29044 (7)	0.23054 (12)	0.0119 (3)	
Ca5	0.41628 (8)	0.49350 (7)	0.68142 (11)	0.0165 (3)	
Ca6	0.41167 (8)	0.15870 (8)	0.70426 (13)	0.0227 (3)	
Mn1	0.25209 (6)	0.46201 (6)	0.50350 (8)	0.0086 (2)	
Mn2	0.24576 (6)	0.20322 (5)	0.29198 (8)	0.0083 (2)	
Mn3	0.24562 (6)	0.22294 (5)	0.79390 (8)	0.00849 (19)	
Mn4	0.24453 (7)	0.04194 (5)	0.27166 (8)	0.00814 (19)	
Mn5	0.24564 (6)	0.05107 (5)	0.76962 (8)	0.00691 (19)	
Mn6	0.25012 (6)	0.37523 (5)	0.27271 (8)	0.00717 (19)	
Mn7	0.25291 (6)	0.38379 (5)	0.77123 (8)	0.00791 (19)	
Mn8	0.25065 (6)	0.29440 (5)	0.03965 (7)	0.0095 (2)	
Mn9	0.23926 (6)	0.12877 (5)	0.53689 (7)	0.0081 (2)	
P1	0.10655 (11)	0.28822 (8)	0.18234 (14)	0.0097 (3)	
P2	0.10885 (11)	0.46008 (8)	0.64796 (14)	0.0081 (3)	
P3	0.10188 (10)	0.14070 (8)	0.68711 (14)	0.0076 (3)	
P4	0.39169 (10)	0.46640 (8)	0.35583 (14)	0.0081 (3)	
P5	0.38516 (11)	0.12096 (8)	0.39767 (15)	0.0090 (3)	
P6	0.39221 (11)	0.30491 (9)	0.89494 (14)	0.0108 (3)	
P7	0.20597 (11)	0.45712 (8)	0.01054 (14)	0.0089 (3)	
P8	0.28687 (10)	0.12488 (8)	0.04287 (13)	0.0126 (3)	
P9	0.28811 (10)	0.29252 (8)	0.54105 (14)	0.0127 (3)	
O1	0.0210 (3)	0.2875 (2)	0.1690 (4)	0.0178 (10)	
O2	0.1379 (3)	0.2231 (2)	0.2543 (4)	0.0135 (10)	
O3	0.1394 (3)	0.3527 (2)	0.2542 (4)	0.0135 (10)	
O4	0.1381 (3)	0.2862 (2)	0.0600 (4)	0.0132 (10)	
O5	0.0237 (3)	0.4560 (2)	0.6302 (4)	0.0152 (10)	
O6	0.1364 (3)	0.5263 (2)	0.7164 (4)	0.0131 (10)	
O7	0.1426 (3)	0.3972 (2)	0.7196 (4)	0.0110 (9)	
O8	0.1411 (3)	0.4594 (2)	0.5248 (4)	0.0135 (10)	
O9	0.0155 (3)	0.1437 (2)	0.6814 (4)	0.0148 (10)	
O10	0.1370 (3)	0.2045 (2)	0.7596 (4)	0.0134 (10)	
O11	0.1339 (3)	0.0742 (2)	0.7528 (4)	0.0116 (9)	
O12	0.1277 (3)	0.1421 (2)	0.5601 (4)	0.0112 (9)	
O13	0.4788 (3)	0.4702 (2)	0.3630 (4)	0.0145 (10)	
O14	0.3614 (3)	0.3989 (2)	0.2923 (4)	0.0090 (9)	
O15	0.3540 (3)	0.5296 (2)	0.2856 (4)	0.0111 (10)	
O16	0.3643 (3)	0.4666 (2)	0.4812 (4)	0.0124 (9)	
O17	0.4724 (3)	0.1198 (3)	0.4171 (4)	0.0168 (11)	
O18	0.3551 (3)	0.1868 (2)	0.3275 (4)	0.0082 (9)	
O19	0.3541 (3)	0.0575 (2)	0.3226 (4)	0.0088 (9)	
O20	0.3502 (3)	0.1198 (2)	0.5191 (4)	0.0124 (10)	
O21	0.4794 (3)	0.3056 (2)	0.9025 (5)	0.0190 (11)	
O22	0.3572 (3)	0.2402 (2)	0.8253 (4)	0.0114 (9)	
O23	0.3593 (3)	0.3692 (2)	0.8256 (4)	0.0117 (10)	
O24	0.3642 (3)	0.3047 (2)	0.0216 (4)	0.0157 (10)	
O25	0.1216 (3)	0.4394 (2)	0.9774 (4)	0.0171 (10)	
O26	0.2491 (3)	0.3998 (2)	0.0859 (4)	0.0129 (10)	
O27	0.2220 (3)	0.5242 (2)	0.0796 (4)	0.0134 (10)	
O28	0.2486 (3)	0.4628 (2)	0.8960 (4)	0.0152 (10)	
O29	0.3736 (3)	0.1122 (3)	0.0715 (4)	0.0210 (11)	
O30	0.2470 (3)	0.0691 (2)	0.9604 (4)	0.0141 (9)	
O31	0.2625 (3)	0.1932 (2)	0.9804 (4)	0.0215 (10)	
O32	0.2460 (3)	0.1243 (2)	0.1567 (4)	0.0168 (9)	
O33	0.3751 (3)	0.2796 (3)	0.5684 (4)	0.0242 (11)	
O34	0.2656 (3)	0.3556 (2)	0.4631 (4)	0.0167 (10)	
O35	0.2452 (3)	0.2315 (2)	0.4746 (4)	0.0159 (9)	
O36	0.2496 (3)	0.3018 (2)	0.6558 (4)	0.0156 (9)	
O37	0.2128 (3)	0.1183 (2)	0.3716 (4)	0.0155 (10)	
O38	0.2705 (3)	0.1411 (2)	0.7083 (4)	0.0094 (9)	
O39	0.2767 (3)	0.2855 (2)	0.2121 (4)	0.0085 (9)	
O40	0.2210 (3)	0.3076 (2)	0.8749 (4)	0.0123 (9)	
O41	0.2217 (3)	0.4622 (2)	0.3310 (4)	0.0105 (9)	
O42	0.2790 (3)	0.4644 (2)	0.6681 (4)	0.0144 (10)	
Ow1	0.4965 (3)	0.2707 (2)	0.4139 (4)	0.0188 (11)	
Ow2	0.4817 (3)	0.3812 (2)	0.6588 (4)	0.0243 (13)	
Ow3	0.4978 (3)	0.0622 (2)	0.6478 (4)	0.0163 (10)	
Ow4	0.4916 (3)	0.1935 (2)	0.1817 (4)	0.0175 (11)	
Ow5	0.5066 (3)	0.4716 (2)	0.8740 (4)	0.0161 (10)	
Ow6	0.4881 (4)	0.1223 (2)	0.8902 (4)	0.0232 (12)	
Ow7	0.4218 (4)	0.4531 (2)	0.0558 (5)	0.0209 (11)	
Ow8	0.5761 (4)	0.3807 (3)	0.4894 (5)	0.0324 (14)	
Ow9	0.5819 (4)	0.2115 (3)	0.0122 (5)	0.0358 (15)	

### Atomic displacement parameters (Å^2^)

**Table:**

	*U*^11^	*U*^22^	*U*^33^	*U*^12^	*U*^13^	*U*^23^
Ca1	0.0091 (6)	0.0167 (6)	0.0148 (6)	−0.0031 (5)	−0.0010 (5)	0.0005 (5)
Ca2	0.0095 (6)	0.0146 (6)	0.0129 (5)	0.0000 (5)	0.0001 (5)	−0.0005 (5)
Ca3	0.0098 (6)	0.0196 (6)	0.0236 (6)	0.0037 (5)	−0.0009 (5)	−0.0084 (5)
Ca4	0.0088 (6)	0.0119 (6)	0.0145 (6)	0.0011 (5)	−0.0008 (5)	−0.0008 (5)
Ca5	0.0086 (6)	0.0254 (7)	0.0155 (6)	−0.0027 (5)	0.0013 (5)	−0.0090 (5)
Ca6	0.0070 (6)	0.0360 (8)	0.0246 (7)	−0.0014 (6)	0.0000 (5)	−0.0201 (6)
Mn1	0.0078 (5)	0.0103 (4)	0.0073 (4)	−0.0007 (4)	−0.0009 (3)	−0.0002 (3)
Mn2	0.0077 (5)	0.0054 (4)	0.0113 (4)	−0.0002 (4)	−0.0012 (4)	0.0006 (3)
Mn3	0.0076 (5)	0.0058 (4)	0.0116 (4)	0.0015 (4)	−0.0010 (4)	−0.0019 (4)
Mn4	0.0079 (5)	0.0077 (4)	0.0084 (4)	−0.0001 (4)	−0.0003 (3)	0.0004 (4)
Mn5	0.0075 (5)	0.0058 (4)	0.0075 (4)	0.0007 (4)	0.0010 (3)	0.0000 (3)
Mn6	0.0065 (5)	0.0060 (4)	0.0087 (4)	0.0008 (4)	−0.0002 (3)	−0.0004 (3)
Mn7	0.0084 (5)	0.0066 (4)	0.0084 (4)	0.0005 (4)	−0.0005 (3)	−0.0013 (3)
Mn8	0.0098 (5)	0.0082 (5)	0.0092 (4)	0.0021 (3)	−0.0038 (4)	−0.0029 (3)
Mn9	0.0075 (5)	0.0077 (4)	0.0090 (4)	−0.0006 (3)	−0.0002 (3)	0.0016 (3)
P1	0.0081 (8)	0.0072 (7)	0.0131 (7)	−0.0006 (6)	−0.0024 (6)	0.0020 (6)
P2	0.0069 (7)	0.0096 (7)	0.0073 (6)	−0.0018 (6)	−0.0011 (5)	0.0006 (5)
P3	0.0061 (7)	0.0058 (7)	0.0107 (6)	0.0007 (5)	0.0003 (6)	−0.0006 (5)
P4	0.0067 (7)	0.0086 (7)	0.0086 (6)	0.0009 (6)	−0.0007 (5)	−0.0009 (5)
P5	0.0086 (8)	0.0060 (7)	0.0120 (7)	−0.0002 (6)	−0.0001 (6)	0.0007 (6)
P6	0.0093 (8)	0.0103 (7)	0.0122 (7)	0.0023 (6)	−0.0015 (6)	−0.0047 (6)
P7	0.0105 (9)	0.0081 (7)	0.0081 (6)	−0.0008 (6)	0.0012 (6)	−0.0008 (6)
P8	0.0108 (8)	0.0156 (8)	0.0112 (7)	−0.0001 (6)	−0.0004 (6)	−0.0038 (6)
P9	0.0098 (8)	0.0152 (7)	0.0125 (7)	−0.0012 (6)	−0.0005 (6)	0.0020 (6)
O1	0.010 (2)	0.014 (2)	0.028 (3)	0.0019 (18)	−0.0028 (19)	0.0032 (18)
O2	0.013 (2)	0.006 (2)	0.021 (2)	0.0005 (17)	−0.0004 (19)	0.0025 (16)
O3	0.012 (2)	0.006 (2)	0.022 (2)	−0.0023 (17)	0.0002 (18)	−0.0019 (16)
O4	0.009 (2)	0.016 (2)	0.014 (2)	−0.0021 (17)	−0.0033 (17)	0.0022 (16)
O5	0.010 (2)	0.017 (2)	0.019 (2)	0.0008 (18)	0.0036 (19)	−0.0007 (18)
O6	0.013 (3)	0.009 (2)	0.016 (2)	0.0003 (17)	−0.0028 (18)	0.0007 (16)
O7	0.012 (2)	0.0086 (19)	0.0120 (19)	−0.0049 (16)	−0.0013 (17)	0.0003 (15)
O8	0.009 (2)	0.018 (2)	0.012 (2)	−0.0045 (17)	−0.0041 (17)	−0.0030 (16)
O9	0.009 (2)	0.014 (2)	0.021 (2)	−0.0013 (18)	−0.0010 (18)	−0.0009 (17)
O10	0.010 (2)	0.012 (2)	0.018 (2)	0.0022 (17)	0.0026 (18)	−0.0027 (16)
O11	0.013 (2)	0.008 (2)	0.015 (2)	0.0011 (17)	0.0040 (18)	0.0020 (16)
O12	0.007 (2)	0.015 (2)	0.0115 (19)	−0.0003 (17)	−0.0004 (16)	0.0048 (16)
O13	0.006 (2)	0.016 (2)	0.022 (2)	−0.0022 (17)	0.0015 (18)	−0.0011 (17)
O14	0.004 (2)	0.011 (2)	0.0118 (19)	0.0009 (16)	−0.0015 (16)	−0.0052 (15)
O15	0.008 (2)	0.011 (2)	0.014 (2)	0.0006 (16)	−0.0025 (17)	0.0037 (16)
O16	0.013 (2)	0.0106 (19)	0.0125 (19)	0.0006 (16)	−0.0018 (17)	0.0000 (16)
O17	0.006 (2)	0.022 (3)	0.022 (2)	0.0027 (19)	0.0000 (19)	0.0058 (19)
O18	0.006 (2)	0.0079 (19)	0.0111 (18)	−0.0001 (16)	0.0010 (16)	0.0026 (15)
O19	0.008 (2)	0.0077 (19)	0.0109 (19)	−0.0005 (16)	0.0006 (16)	−0.0032 (15)
O20	0.014 (2)	0.014 (2)	0.0096 (19)	−0.0039 (18)	0.0027 (17)	0.0004 (16)
O21	0.007 (2)	0.018 (2)	0.031 (3)	0.0003 (18)	−0.0004 (19)	−0.0054 (19)
O22	0.009 (2)	0.010 (2)	0.015 (2)	−0.0006 (16)	−0.0016 (17)	−0.0078 (16)
O23	0.010 (2)	0.012 (2)	0.0131 (19)	0.0044 (16)	0.0000 (17)	0.0006 (15)
O24	0.020 (3)	0.018 (2)	0.0088 (19)	0.0033 (19)	−0.0033 (17)	−0.0060 (16)
O25	0.016 (3)	0.015 (2)	0.021 (2)	−0.001 (2)	0.003 (2)	−0.0009 (18)
O26	0.019 (3)	0.011 (2)	0.0078 (19)	0.0009 (19)	−0.0038 (18)	−0.0015 (17)
O27	0.018 (2)	0.009 (2)	0.012 (2)	−0.0071 (17)	−0.0014 (18)	−0.0016 (15)
O28	0.019 (3)	0.016 (2)	0.011 (2)	−0.002 (2)	0.0075 (19)	−0.0013 (18)
O29	0.008 (2)	0.026 (2)	0.027 (3)	0.0065 (19)	−0.0070 (19)	−0.009 (2)
O30	0.014 (2)	0.014 (2)	0.014 (2)	−0.0024 (17)	0.0003 (17)	−0.0057 (16)
O31	0.026 (3)	0.016 (2)	0.023 (2)	−0.0009 (19)	0.002 (2)	0.0011 (19)
O32	0.014 (2)	0.023 (2)	0.014 (2)	0.0051 (18)	−0.0009 (17)	−0.0058 (17)
O33	0.010 (2)	0.034 (3)	0.027 (3)	0.003 (2)	−0.005 (2)	−0.004 (2)
O34	0.027 (3)	0.012 (2)	0.0115 (19)	−0.0068 (18)	0.0014 (18)	0.0029 (15)
O35	0.014 (2)	0.012 (2)	0.021 (2)	−0.0047 (17)	0.0007 (18)	0.0015 (17)
O36	0.011 (2)	0.018 (2)	0.017 (2)	0.0004 (17)	0.0024 (17)	0.0048 (17)
O37	0.013 (2)	0.018 (2)	0.014 (2)	−0.0048 (18)	−0.0029 (18)	0.0045 (17)
O38	0.007 (2)	0.0105 (19)	0.0115 (19)	−0.0017 (16)	0.0019 (16)	−0.0034 (16)
O39	0.009 (2)	0.0048 (17)	0.0112 (18)	−0.0014 (15)	−0.0012 (16)	−0.0004 (15)
O40	0.012 (2)	0.010 (2)	0.0134 (19)	0.0045 (17)	−0.0019 (17)	−0.0073 (16)
O41	0.011 (2)	0.0119 (19)	0.0090 (18)	−0.0010 (17)	0.0009 (17)	0.0032 (15)
O42	0.008 (2)	0.027 (2)	0.0075 (18)	−0.0011 (18)	−0.0023 (17)	−0.0029 (17)
Ow1	0.027 (3)	0.014 (2)	0.014 (2)	0.004 (2)	−0.004 (2)	−0.0036 (18)
Ow2	0.027 (3)	0.019 (2)	0.025 (2)	0.002 (2)	−0.004 (2)	−0.0063 (19)
Ow3	0.018 (2)	0.017 (2)	0.012 (2)	−0.001 (2)	−0.0034 (18)	−0.0009 (18)
Ow4	0.022 (3)	0.015 (2)	0.014 (2)	0.003 (2)	−0.0015 (19)	−0.0037 (17)
Ow5	0.019 (3)	0.014 (2)	0.016 (2)	0.001 (2)	0.0055 (19)	−0.0038 (17)
Ow6	0.032 (3)	0.021 (3)	0.017 (2)	0.001 (2)	0.004 (2)	−0.0049 (19)
Ow7	0.026 (3)	0.014 (2)	0.024 (2)	−0.005 (2)	0.008 (2)	−0.0063 (19)
Ow8	0.038 (4)	0.028 (3)	0.030 (3)	−0.011 (2)	0.004 (3)	−0.008 (2)
Ow9	0.048 (4)	0.026 (3)	0.036 (3)	−0.018 (3)	0.014 (3)	−0.010 (2)

### Geometric parameters (Å, º)

**Table:**

Ca1—O13^i^	2.296 (5)	Mn4—O42^viii^	2.038 (5)
Ca1—Ow3	2.348 (5)	Mn4—O32	2.062 (5)
Ca1—O8^ii^	2.396 (5)	Mn4—O28^viii^	2.079 (5)
Ca1—O41^ii^	2.437 (5)	Mn5—O15^i^	1.913 (5)
Ca1—Ow6	2.467 (6)	Mn5—O41^i^	1.924 (4)
Ca1—O3^ii^	2.508 (5)	Mn5—O38	1.949 (4)
Ca1—O11^iii^	2.540 (5)	Mn5—O11	1.976 (5)
Ca2—O17	2.261 (5)	Mn5—O30	2.178 (4)
Ca2—O4^ii^	2.420 (5)	Mn5—O27^i^	2.199 (5)
Ca2—O40^iv^	2.449 (5)	Mn6—O41	1.901 (5)
Ca2—O7^iv^	2.469 (5)	Mn6—O39	1.949 (4)
Ca2—Ow4	2.507 (5)	Mn6—O3	1.957 (5)
Ca2—Ow1	2.525 (5)	Mn6—O14	1.971 (5)
Ca2—O25^iv^	2.531 (5)	Mn6—O26	2.158 (5)
Ca3—O21	2.270 (5)	Mn6—O34	2.166 (4)
Ca3—Ow5	2.335 (5)	Mn7—O23	1.898 (5)
Ca3—O37^ii^	2.393 (6)	Mn7—O7	1.952 (5)
Ca3—O2^ii^	2.394 (5)	Mn7—O40	2.004 (5)
Ca3—Ow2	2.445 (5)	Mn7—O42	2.034 (5)
Ca3—O12^ii^	2.576 (4)	Mn7—O36	2.055 (4)
Ca3—O6^v^	2.710 (5)	Mn7—O28	2.090 (5)
Ca3—Mn2^ii^	3.4002 (18)	Mn8—O40^ix^	1.888 (4)
Ca3—Mn9^ii^	3.4199 (17)	Mn8—O39	1.953 (4)
Ca3—P6	3.540 (2)	Mn8—O4	1.997 (5)
Ca3—Mn4^ii^	3.5519 (19)	Mn8—O24	2.013 (5)
Ca3—Ca5	3.896 (2)	Mn8—O31^ix^	2.096 (5)
Ca4—O9^iv^	2.271 (6)	Mn8—O26	2.116 (5)
Ca4—Ow1	2.389 (5)	Mn9—O37	1.878 (4)
Ca4—Ow4	2.398 (5)	Mn9—O38	1.961 (4)
Ca4—O39	2.400 (5)	Mn9—O20	1.964 (5)
Ca4—O24	2.438 (5)	Mn9—O12	1.999 (5)
Ca4—O14	2.444 (5)	Mn9—O27^i^	2.119 (4)
Ca4—O18	2.575 (5)	Mn9—O35	2.125 (4)
Ca5—O5^v^	2.240 (5)	P1—O1	1.474 (6)
Ca5—O19^vi^	2.375 (5)	P1—O4	1.542 (5)
Ca5—O16	2.391 (5)	P1—O3	1.565 (5)
Ca5—O42	2.434 (5)	P1—O2	1.567 (5)
Ca5—Ow2	2.488 (5)	P2—O5	1.469 (6)
Ca5—Ow5	2.562 (5)	P2—O7	1.542 (5)
Ca5—O29^vi^	2.684 (5)	P2—O6	1.549 (5)
Ca6—O1^ii^	2.241 (5)	P2—O8	1.557 (5)
Ca6—O20	2.357 (5)	P3—O9	1.492 (6)
Ca6—O22	2.359 (5)	P3—O12	1.549 (5)
Ca6—Ow6	2.452 (5)	P3—O11	1.561 (5)
Ca6—O38	2.478 (5)	P3—O10	1.570 (5)
Ca6—Ow3	2.526 (5)	P4—O13	1.506 (6)
Ca6—O33	2.838 (5)	P4—O16	1.542 (5)
Ca6—O15^i^	2.892 (5)	P4—O14	1.558 (5)
Mn1—O42	1.862 (4)	P4—O15	1.563 (5)
Mn1—O41	1.956 (4)	P5—O17	1.503 (5)
Mn1—O8	1.967 (5)	P5—O19	1.558 (5)
Mn1—O16	1.992 (5)	P5—O20	1.561 (5)
Mn1—O34	2.137 (4)	P5—O18	1.563 (5)
Mn1—O30^vii^	2.138 (4)	P6—O21	1.505 (5)
Mn2—O2	1.909 (5)	P6—O23	1.547 (5)
Mn2—O18	1.919 (5)	P6—O24^x^	1.561 (5)
Mn2—O39	1.942 (4)	P6—O22	1.568 (5)
Mn2—O37	1.994 (5)	P7—O25^ix^	1.507 (6)
Mn2—O35	2.133 (5)	P7—O27	1.528 (5)
Mn2—O32	2.164 (4)	P7—O26	1.542 (5)
Mn3—O10	1.913 (5)	P7—O28^ix^	1.564 (5)
Mn3—O38	1.936 (4)	P8—O29	1.521 (5)
Mn3—O40	1.954 (4)	P8—O32	1.537 (5)
Mn3—O22	1.956 (5)	P8—O30^ix^	1.540 (4)
Mn3—O31	2.168 (5)	P8—O31^ix^	1.540 (5)
Mn3—O36	2.192 (4)	P9—O33	1.525 (5)
Mn4—O6^viii^	1.931 (5)	P9—O34	1.534 (4)
Mn4—O19	1.944 (5)	P9—O36	1.534 (5)
Mn4—O37	1.981 (5)	P9—O35	1.547 (4)
			
O42—Mn1—O41	178.1 (2)	O40—Mn7—O42	176.3 (2)
O42—Mn1—O8	90.9 (2)	O23—Mn7—O36	92.47 (19)
O41—Mn1—O8	88.1 (2)	O7—Mn7—O36	87.76 (18)
O42—Mn1—O16	89.4 (2)	O40—Mn7—O36	78.86 (17)
O41—Mn1—O16	91.7 (2)	O42—Mn7—O36	103.18 (18)
O8—Mn1—O16	178.9 (2)	O23—Mn7—O28	90.1 (2)
O42—Mn1—O34	102.50 (19)	O7—Mn7—O28	89.6 (2)
O41—Mn1—O34	79.25 (17)	O40—Mn7—O28	96.68 (18)
O8—Mn1—O34	97.7 (2)	O42—Mn7—O28	81.14 (17)
O16—Mn1—O34	83.30 (19)	O36—Mn7—O28	174.9 (2)
O42—Mn1—O30^vii^	101.58 (19)	O40^ix^—Mn8—O39	176.3 (2)
O41—Mn1—O30^vii^	76.85 (17)	O40^ix^—Mn8—O4	88.0 (2)
O8—Mn1—O30^vii^	92.17 (19)	O39—Mn8—O4	89.7 (2)
O16—Mn1—O30^vii^	86.73 (18)	O40^ix^—Mn8—O24	92.6 (2)
O34—Mn1—O30^vii^	153.77 (17)	O39—Mn8—O24	89.62 (19)
O2—Mn2—O18	177.7 (2)	O4—Mn8—O24	178.60 (19)
O2—Mn2—O39	92.6 (2)	O40^ix^—Mn8—O31^ix^	80.77 (18)
O18—Mn2—O39	85.1 (2)	O39—Mn8—O31^ix^	102.39 (18)
O2—Mn2—O37	86.8 (2)	O4—Mn8—O31^ix^	95.48 (19)
O18—Mn2—O37	95.5 (2)	O24—Mn8—O31^ix^	85.9 (2)
O39—Mn2—O37	179.1 (2)	O40^ix^—Mn8—O26	95.79 (18)
O2—Mn2—O35	92.82 (19)	O39—Mn8—O26	81.27 (17)
O18—Mn2—O35	87.48 (18)	O4—Mn8—O26	90.5 (2)
O39—Mn2—O35	105.53 (17)	O24—Mn8—O26	88.2 (2)
O37—Mn2—O35	75.24 (17)	O31^ix^—Mn8—O26	173.0 (2)
O2—Mn2—O32	93.90 (19)	O37—Mn9—O38	177.9 (2)
O18—Mn2—O32	86.98 (18)	O37—Mn9—O20	91.1 (2)
O39—Mn2—O32	103.55 (18)	O38—Mn9—O20	87.09 (19)
O37—Mn2—O32	75.77 (18)	O37—Mn9—O12	90.9 (2)
O35—Mn2—O32	149.79 (17)	O38—Mn9—O12	90.9 (2)
O10—Mn3—O38	90.9 (2)	O20—Mn9—O12	177.1 (2)
O10—Mn3—O40	89.4 (2)	O37—Mn9—O27^i^	95.57 (19)
O38—Mn3—O40	177.80 (19)	O38—Mn9—O27^i^	85.62 (17)
O10—Mn3—O22	178.4 (2)	O20—Mn9—O27^i^	95.9 (2)
O38—Mn3—O22	87.6 (2)	O12—Mn9—O27^i^	86.0 (2)
O40—Mn3—O22	92.1 (2)	O37—Mn9—O35	77.82 (18)
O10—Mn3—O31	99.5 (2)	O38—Mn9—O35	101.11 (18)
O38—Mn3—O31	104.59 (18)	O20—Mn9—O35	87.99 (19)
O40—Mn3—O31	77.50 (18)	O12—Mn9—O35	90.35 (18)
O22—Mn3—O31	81.37 (19)	O27^i^—Mn9—O35	172.40 (18)
O10—Mn3—O36	95.68 (18)	O1—P1—O4	111.4 (3)
O38—Mn3—O36	101.16 (17)	O1—P1—O3	111.3 (3)
O40—Mn3—O36	76.65 (17)	O4—P1—O3	109.8 (3)
O22—Mn3—O36	84.06 (18)	O1—P1—O2	109.3 (3)
O31—Mn3—O36	149.72 (17)	O4—P1—O2	107.8 (3)
O6^viii^—Mn4—O19	178.4 (2)	O3—P1—O2	107.2 (3)
O6^viii^—Mn4—O37	88.8 (2)	O5—P2—O7	110.0 (3)
O19—Mn4—O37	92.4 (2)	O5—P2—O6	111.1 (3)
O6^viii^—Mn4—O42^viii^	92.1 (2)	O7—P2—O6	108.7 (3)
O19—Mn4—O42^viii^	86.7 (2)	O5—P2—O8	109.7 (3)
O37—Mn4—O42^viii^	178.9 (2)	O7—P2—O8	107.9 (3)
O6^viii^—Mn4—O32	90.06 (19)	O6—P2—O8	109.3 (3)
O19—Mn4—O32	89.03 (19)	O9—P3—O12	110.8 (3)
O37—Mn4—O32	78.44 (18)	O9—P3—O11	110.7 (3)
O42^viii^—Mn4—O32	100.86 (18)	O12—P3—O11	109.1 (3)
O6^viii^—Mn4—O28^viii^	93.3 (2)	O9—P3—O10	108.5 (3)
O19—Mn4—O28^viii^	87.7 (2)	O12—P3—O10	109.4 (3)
O37—Mn4—O28^viii^	99.29 (19)	O11—P3—O10	108.2 (3)
O42^viii^—Mn4—O28^viii^	81.34 (18)	O13—P4—O16	111.3 (3)
O32—Mn4—O28^viii^	175.9 (2)	O13—P4—O14	110.4 (3)
O15^i^—Mn5—O41^i^	91.2 (2)	O16—P4—O14	107.5 (3)
O15^i^—Mn5—O38	88.6 (2)	O13—P4—O15	110.4 (3)
O41^i^—Mn5—O38	179.6 (3)	O16—P4—O15	107.9 (3)
O15^i^—Mn5—O11	179.5 (2)	O14—P4—O15	109.3 (3)
O41^i^—Mn5—O11	89.4 (2)	O17—P5—O19	110.2 (3)
O38—Mn5—O11	90.9 (2)	O17—P5—O20	111.0 (3)
O15^i^—Mn5—O30	92.51 (18)	O19—P5—O20	108.7 (3)
O41^i^—Mn5—O30	76.54 (17)	O17—P5—O18	110.9 (3)
O38—Mn5—O30	103.22 (17)	O19—P5—O18	107.5 (3)
O11—Mn5—O30	87.52 (18)	O20—P5—O18	108.4 (3)
O15^i^—Mn5—O27^i^	96.3 (2)	O21—P6—O23	109.3 (3)
O41^i^—Mn5—O27^i^	96.52 (17)	O21—P6—O24^x^	111.4 (3)
O38—Mn5—O27^i^	83.76 (17)	O23—P6—O24^x^	109.2 (3)
O11—Mn5—O27^i^	83.68 (19)	O21—P6—O22	111.3 (3)
O30—Mn5—O27^i^	168.88 (19)	O23—P6—O22	107.3 (3)
O41—Mn6—O39	178.6 (3)	O24^x^—P6—O22	108.2 (3)
O41—Mn6—O3	86.9 (2)	O25^ix^—P7—O27	115.8 (3)
O39—Mn6—O3	91.8 (2)	O25^ix^—P7—O26	111.5 (3)
O41—Mn6—O14	92.5 (2)	O27—P7—O26	106.8 (3)
O39—Mn6—O14	88.88 (19)	O25^ix^—P7—O28^ix^	110.4 (3)
O3—Mn6—O14	179.3 (2)	O27—P7—O28^ix^	106.6 (3)
O41—Mn6—O26	99.65 (18)	O26—P7—O28^ix^	105.1 (3)
O39—Mn6—O26	80.28 (17)	O29—P8—O32	111.2 (3)
O3—Mn6—O26	92.8 (2)	O29—P8—O30^ix^	112.2 (3)
O14—Mn6—O26	87.44 (19)	O32—P8—O30^ix^	106.3 (3)
O41—Mn6—O34	79.70 (17)	O29—P8—O31^ix^	116.7 (3)
O39—Mn6—O34	100.54 (17)	O32—P8—O31^ix^	105.1 (3)
O3—Mn6—O34	94.25 (19)	O30^ix^—P8—O31^ix^	104.6 (3)
O14—Mn6—O34	85.51 (19)	O33—P9—O34	115.3 (3)
O26—Mn6—O34	172.9 (2)	O33—P9—O36	111.4 (3)
O23—Mn7—O7	178.2 (2)	O34—P9—O36	106.5 (3)
O23—Mn7—O40	91.4 (2)	O33—P9—O35	112.4 (3)
O7—Mn7—O40	86.90 (19)	O34—P9—O35	105.0 (2)
O23—Mn7—O42	91.6 (2)	O36—P9—O35	105.5 (3)
O7—Mn7—O42	90.1 (2)		

Symmetry codes: (i) *x*, *y*−1/2, *z*+1/2; (ii) *x*+1/2, −*y*+1/2, *z*+1/2; (iii) *x*+1/2, −*y*, *z*; (iv) *x*+1/2, −*y*+1/2, *z*−1/2; (v) *x*+1/2, −*y*+1, *z*; (vi) *x*, *y*+1/2, *z*+1/2; (vii) *x*, *y*+1/2, *z*−1/2; (viii) *x*, *y*−1/2, *z*−1/2; (ix) *x*, *y*, *z*−1; (x) *x*, *y*, *z*+1.

### Hydrogen-bond geometry (Å)

**Table:**

*D*—H···*A*	*D*···*A*
O*w*1···O17	2.964 (7)
O*w*1···O33	2.889 (8)
O*w*2···O33	2.815 (7)
O*w*2···O*w*8	2.655 (8)
O*w*3···O1^ii^	2.957 (4)
O*w*3···O17	2.821 (6)
O*w*4···O29	2.764 (7)
O*w*4···O*w*9	2.631 (8)
O*w*5···O*w*7^x^	2.682 (8)
O*w*5···O25^v^	2.792 (7)
O*w*6···O13^i^	2.977 (6)
O*w*6···O*w*9^x^	2.651 (7)
O*w*7···O9^iv^	2.768 (7)
O*w*7···O*w*3^vii^	2.645 (7)
O*w*8···O13	2.710 (8)
O*w*8···O*w*1	2.635 (7)
O*w*9···O12^iv^	2.989 (7)
O*w*9···O21^ix^	2.743 (8)

Symmetry codes: (i) *x*, *y*−1/2, *z*+1/2; (ii) *x*+1/2, −*y*+1/2, *z*+1/2; (iv) *x*+1/2, −*y*+1/2, *z*−1/2; (v) *x*+1/2, −*y*+1, *z*; (vii) *x*, *y*+1/2, *z*−1/2; (ix) *x*, *y*, *z*−1; (x) *x*, *y*, *z*+1.
